# Editorial: RNA Diseases in Humans—From Fundamental Research to Therapeutic Applications

**DOI:** 10.3389/fmolb.2019.00053

**Published:** 2019-07-16

**Authors:** Naoyuki Kataoka, Akila Mayeda, Kinji Ohno

**Affiliations:** ^1^Laboratory of Cell Regulation, Departments of Applied Animal Sciences and Applied Biological Chemistry, Graduate School of Agriculture and Life Sciences, The University of Tokyo, Tokyo, Japan; ^2^Division of Gene Expression Mechanism, Institute for Comprehensive Medical Science, Fujita Health University, Toyoake, Japan; ^3^Division of Neurogenetics, Center for Neurological Diseases and Cancer, Nagoya University Graduate School of Medicine, Nagoya, Japan

**Keywords:** RNA, RNA disease, splicing, non-coding RNA (ncRNA), translation, virus

In higher eukaryotes, many different RNAs are encoded in and transcribed from genomic DNAs. Transcribed RNA molecules undergo multiple post-transcriptional processes such as splicing, editing, modification, translation, and degradation. A defect, mis-regulation, or malfunction of these processes often results in diseases in humans, and recently they have been referred to as “RNA diseases” (Scotti and Swanson, 2016; Kataoka, 2017; Ohno et al., 2018). There is an increasing number of studies focused on RNA diseases, which are aimed at unraveling the fundamental molecular mechanisms and developing therapeutic approaches. The goal of this special issue is to introduce RNA diseases to not only RNA scientists but also clinical scientists who are interested in seeking for cures of the diseases.

Pre-mRNA splicing is one of the major processes for post-transcriptional regulatory steps in eukaryotes. Defects in this step result in many diseases, such as neuromuscular diseases and cancers. Splicing takes place in a large multi-RNA-protein complex, called spliceosome. Over one hundred proteins are involved in this step, and many splicing regulatory proteins have been reported. It has been demonstrated that cancer cells undergo cancer-specific splicing patterns. In this special issue on RNA diseases, El Marabti and Younis describe distinct cases of alternative splicing in cancer. They report several categories of splicing aberrations causing alterations in cancer-related genes, mutations in genes encoding splicing factors or core spliceosomal subunits and the disruptions of the balance of RNA-binding proteins. Myelodysplastic syndrome (MDS) is a heterogeneous group of chronic myeloid neoplasms characterized by ineffective hematopoiesis, peripheral blood cytopenia and a high risk of progression to acute myeloid leukemia (AML). Recently it has been shown that mutations on splicing factors are causes of MDS. SRSF2 is one of the major responsible genes for MDS. Masaki et al. found that MDS associated mutations in SRSF2 alter binding affinity to CCWG sequence in RNA and cause aberrant splicing of a subset of genes including EZH2. Estrogen Receptor (ER) α has a critical role in majority of breast cancer. Ohe et al. analyzed the alternative splicing of ERα by using *in vitro* splicing assays and found that HMGA1a induced exon skipping of a shortened exon 1 of ERα. Regulation of ERα alternative splicing by an HMGA1a gives us a novel insight on 5′ splice site regulation by U1 snRNP, as well as a possible target in breast cancer therapy.

Pre-mRNA splicing and associated proteins are also involved in tissue development and diseases. Su et al. describe the importance of alternative splicing during neuronal differentiation. Alternative splicing modulates signaling activity, centriolar dynamics, and metabolic pathways, and it also contributes to neurogenesis and brain development. This review shed light on how splicing defects cause brain disorders and diseases. Among those diseases, one of the most famous disease genes is Fused in sarcoma (FUS). FUS is an RNA binding protein that regulates RNA metabolism including alternative splicing, transcription, and RNA transportation. This protein is genetically and pathologically involved in frontotemporal lobar degeneration (FTLD)/amyotrophic lateral sclerosis (ALS). Ishigaki and Sobue summarize the functions of FUS protein in alternative splicing, transcription, mRNA destabilization, axonal transport, and morphological maintenance of neurons. They conclude that a biological link between loss of FUS function, Tau isoform alteration, aberrant post-synaptic function, and phenotypic expression might lead to the sequential cascade culminating in FTLD. RNA splicing factor is also involved in muscle differentiation and cardiomyopathy. RBM20 is a vertebrate-specific RNA-binding protein, and it has initially been identified as one of dilated cardiomyopathy (DCM)-linked genes. RBM20 is a regulator of heart-specific alternative splicing, and one of the major targets for RBM20 is the titin (*TTN*) gene. As titin is the most important factor for passive tension of cardiomyocytes, extensive heart-specific and developmentally regulated alternative splicing of the *TTN* pre-mRNA by RBM20 plays a critical role in passive stiffness and diastolic function of the heart. Manipulation of the *Ttn* pre-mRNA splicing raises RBM20 as a potential therapeutic target (Watanabe et al.).

Translation of mRNA is another critical step for gene expression. Translation of most mRNAs in cells are mediated by the cap structure at the 5′ terminus. However, translation of some cellular mRNAs and viral mRNAs are cap-independent. Internal ribosome entry site (IRES) can recruit eukaryotic initiation factor (eIF) complex and ribosome subunit on mRNA in a cap-independent manner. Hepatitis A virus (HAV) utilizes IRES-dependent translation, but unlike most viral IRESs, HAV IRES-mediated translation requires eIF4E and the 3′ end of HAV RNA is polyadenylated. Sadahiro et al. analyzed HAV-IRES-mediated translation in a cell-free system derived from either non-hepatic cells or hepatoma cells and revealed that HAV IRES-mediated translation activity in hepatoma cell extracts is higher as compared to extracts derived from a non-hepatic line. Their results strongly suggest that HAV IRES-mediated translation is upregulated by a hepatic cell-specific activator in a poly(A) tail-independent manner.

Recently, it has been well-accepted that long non-coding RNAs (lncRNAs) also play important roles in gene expression in cells. Some of them can be good markers for cancer progression (Yoshimoto et al., 2016). In the manuscript by Tano et al. demonstrated that MALAT1 has a function in repressing the promoter of p53 (TP53) tumor suppressor gene. The p53 targets were upregulated by MALAT1 knockdown in A549 human lung adenocarcinoma cells, which were mediated by transcriptional activation of p53 through MALAT1 depletion. They also identified a minimal MALAT1-responsive region in the P1 promoter of p53 gene. These results suggest that MALAT1 affects the expression of p53 target genes through repressing p53 promoter activity.

On the other hand, the expression of lncRNAs is also regulated by signal cascade. Mullin et al. identified lncRNAs and protein-coding RNAs that are induced by β-catenin activity in mouse dermal fibroblasts. They identified 111 lncRNAs that are differentially expressed in response to activation of Wnt/β-catenin signaling. Among them, two novel Wnt signaling- Induced Non-Coding RNA (Wincr) transcripts, Wincr1 and Wincr2, were validated. These two lncRNAs are highly expressed in mouse embryonic skin and perinatal dermal fibroblasts, and Wincr1 expression levels in perinatal dermal fibroblasts affects the expression of key markers of fibrosis. These results suggest that β-catenin signaling-responsive lncRNAs modulate dermal fibroblast behavior and collagen accumulation in dermal fibrosis, providing nodes for therapeutic intervention.

The lncRNAs are thought to exhibit their functions as RNA-Protein complex. Jalali et al. utilized the available genome-scale experimental datasets of RNA binding proteins (RBP) to understand the role of lncRNAs in terms of its interactions with RBPs. Their analysis suggests that density of interaction sites for the RBPs was significantly higher for specific sub-classes of lncRNAs when compared to protein-coding transcripts. The significant enrichment of RBP sites across some lncRNA classes strongly suggests that these interactions might be important in the functional roles of lncRNA such as silencing, mRNA processing, and transport.

Interestingly, lncRNAs also serve as templates of micropeptides. In the review article by Yeasmin et al. they summarize the current progress and view of micropeptides encoded in putative short open reading frames (sORFs) within transcripts previously identified as lncRNAs or transcripts of unknown function (TUFs). There are several lines of evidence for significant divergent roles of micropeptides in many fundamental biological processes and even important relationships with pathogenesis.

In conclusion, RNA and RNA binding proteins are involved in most of gene expression steps in higher eukaryotes. The mutations in them cause RNA diseases in human. We sincerely hope the increasing numbers of scientific findings including papers in this special issue will shed light on the disease mechanisms and lead to the development of novel therapeutic strategies for them.

## Author Contributions

NK, AM, and KO wrote the paper. NK took the primary responsibility for the final content. NK, AM, and KO read and approved the final manuscript.

### Conflict of Interest Statement

The authors declare that the research was conducted in the absence of any commercial or financial relationships that could be construed as a potential conflict of interest.
